# Distal reentry as a functional primary entry: Insights from blood flow simulation in residual DeBakey IIIb aortic dissection after thoracic endovascular aortic repair

**DOI:** 10.1016/j.xjse.2025.100067

**Published:** 2025-08-29

**Authors:** Yumeka Tamai, Chikara Ueki, Genichi Sakaguchi

**Affiliations:** a Department of Cardiovascular Surgery, Kindai University Hospital, Osakasayama, Osaka, Japan; b Graduate School of Public Health, Shizuoka Graduate University of Public Health, Shizuoka, Shizuoka, Japan

**Figure:**
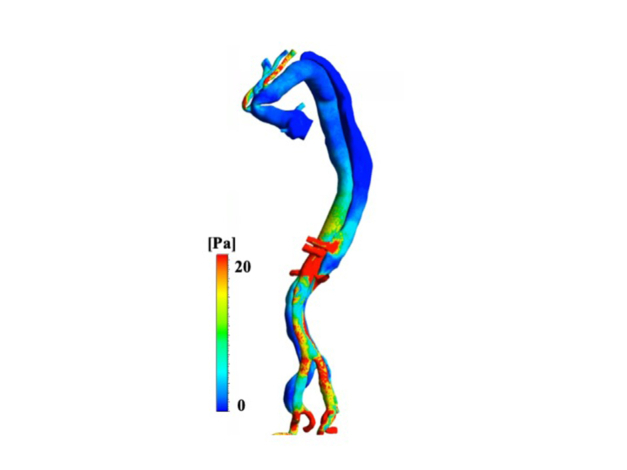
Blood flow via residual reentries changed, and an accompanying elevation of WSS was observed.

**Central Message**

CFD after TEVAR for DeBakey IIIb dissection showed that the most proximal residual reentry can act as a “functional primary entry” due to dynamic changes in blood flow via residual reentries.

Thoracic endovascular aortic repair (TEVAR) is a common intervention for dissecting thoracoabdominal aortic aneurysms; however, long-term aortic dilation remains a concern. We report a case of residual type B aortic dissection treated with 2 TEVARs, where computational fluid dynamics (CFD) highlighted the hemodynamic role of distal reentries.

A 47-year-old man, after total arch replacement for DeBakey type I dissection, had multiple residual reentries. The first TEVAR (3 months later) covered the distal left subclavian artery reentry. While the thoracic aorta was remodeled, the abdominal aorta at the celiac trunk reentry dilated. A second TEVAR (7 months later) targeted this celiac reentry. Despite remodeling of the thoracic false lumen (FL), persistent FL flow via the left renal artery reentry led to continued aortic enlargement (Figure 1).

**Figure 1:**
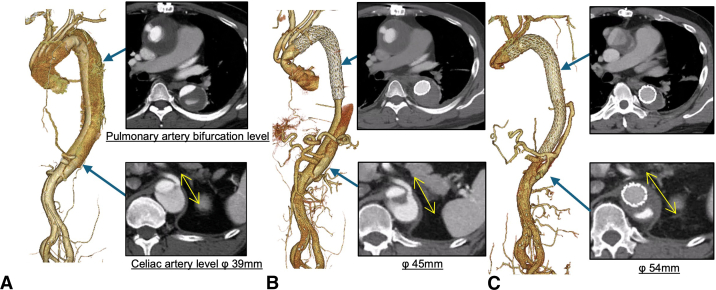
Three-dimensional computed tomography images illustrating FL morphology and axial section at the level of pulmonary artery bifurcation and celiac artery reentry (A) after total arch replacement, (B) after the first TEVAR, (C) and after the second TEVAR. Insets demonstrate progressive abdominal aortic dilatation at the celiac trunk level (diameters: 39 mm [A], 45 mm 5 months after the first TEVAR [B], and 54 mm 9 months after the second TEVAR [C]). Meanwhile, FL in the descending thoracic aorta had remodeled.

The CFD analysis (Table 1) revealed significant hemodynamic changes. After the first TEVAR, the celiac reentry flow reversed into the FL (+62.9 mL/min from −35.0 mL/min before the first TEVAR). Negative values indicate flow from the FL to the true lumen (TL), and positive values indicate flow from the TL to the FL, with an elevated FL wall shear stress (WSS) (Figure 2, *A* and *B*). After the second TEVAR, FL flow via the left renal artery reentry increased significantly (+177.7 mL/min from +77.6 mL/min), with further WSS elevation in the abdominal FL (Figure 2, *C*, and Video 1). In contrast to the elevated WSS, the oscillatory shear index was relatively low in the areas of progressive aortic dilatation at each time point (Figure 3). These findings suggest that proximal reentry closure can transform distal reentries into functional primary entries.

**Table 1:** Mean blood flow (mL/min) through reentries during the computational fluid dynamics analysis

Reentry level	After total arch replacement	After first TEVAR	After second TEVAR
Distal arch	+116.0	-	-
Celiac artery	−35.0	+62.9	-
Left renal artery	+99.6	+77.6	+177.7
Right common iliac artery	−148.0	+38.1	+125.5

Positive values indicate flow from the TL to the FL, and negative values indicate flow from the FL to the TL. *TEVAR,* Thoracic endovascular aortic repair.

**Figure 2:**
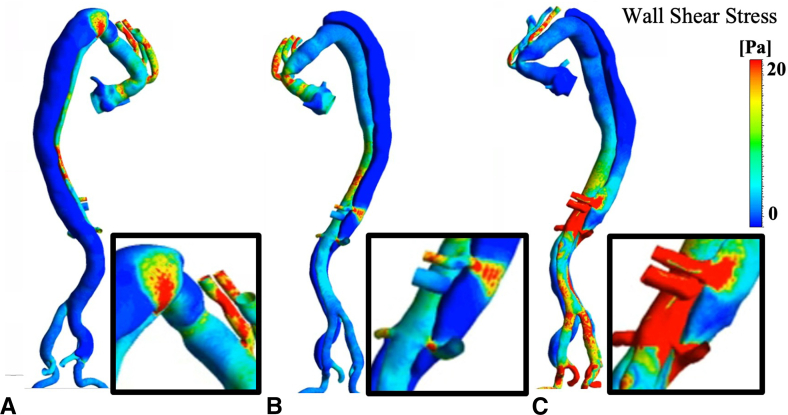
WSS distribution maps corresponding to the time points in Figure 1. Elevated WSS (indicated by *red areas*) is observed along with FL wall at the level of (A) distal arch before TEVAR, (B) celiac reentry after the first TEVAR, and (C) celiac and left renal reentry after the second TEVAR.

**Figure 3:**
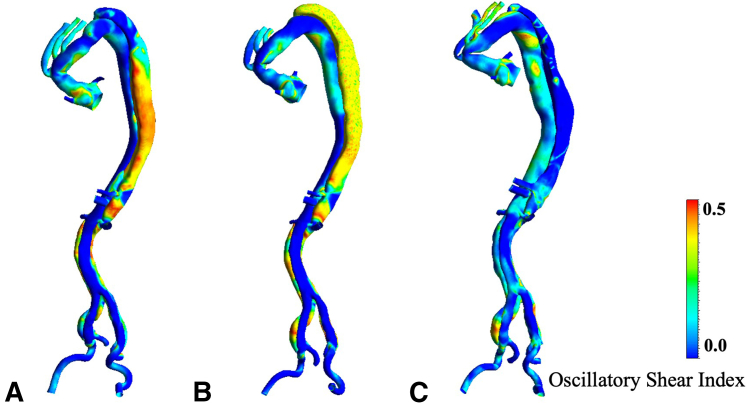
Oscillatory shear index at the same time points as in Figures 1 and 2. Oscillatory shear index showed relatively low values in the FL corresponding to areas with elevated WSS, especially at the celiac reentry level.

## Discussion

This case report illustrates dynamic hemodynamics in residual DeBakey IIIb dissection post-TEVAR. CFD analyses revealed that staged closure of proximal reentries led to sequential shifts in FL perfusion. In our patient, the celiac reentry initially became a new FL inflow source after the first TEVAR; subsequently, the left renal artery reentry assumed this role after the second TEVAR. This CFD-visualized transformation highlights that the most proximal residual reentry can become a “functional primary entry” (a dominant channel for FL inflow), driving persistent FL perfusion and impacting late aortic dilatation.

Managing patent FLs with residual reentries in chronic DeBakey IIIb dissection is challenging. Previous studies on TEVAR for chronic DeBakey IIIb aneurysms confirm that distal residual reentries—especially large ones—are critical risk factors for unfavorable remodeling and late expansion.^1^^,^^2^ Our case, in which the patient has an emergency “functional primary entry” from such tears, aligns with these findings. The observed elevated WSS also correlates with its known role in aneurysmal growth,^3^^,^^4^ emphasizing these adverse hemodynamics.

The “functional primary entry” concept, involving the most proximal residual reentry, has key clinical implications. If dilatation occurs, targeting the “functional primary entry” is a crucial consideration for reintervention.

## Conclusions

In DeBakey IIIb dissection, proximal entry closure can lead to distal reentries transforming into “functional primary entries.” Notably, the most proximal residual reentry can become hemodynamically dominant, driving persistent FL perfusion and late aortic dilatation. Recognizing this dynamic process is crucial for managing these complex cases and guiding reintervention.

The authors thank Cardio Flow Design Inc for analyzing the data.

## Conflict of Interest Statement

The authors reported no conflicts of interest.

The *Journal* policy requires editors and reviewers to disclose conflicts of interest and to decline handling or reviewing manuscripts for which they may have a conflict of interest. The editors and reviewers of this article have no conflicts of interest.

## Supplementary Data

Video 1 Four-dimensional simulation of WSS and blood flow through residual reentries. The video displays the dynamic changes in WSS distribution on the aortic wall throughout a cardiac cycle. It presents 3 consecutive models: (1) after total arch replacement (pre-TEVAR), (2) after the first TEVAR, and (3) after the second TEVAR. The color scale indicates WSS magnitude, with red representing elevated levels. Video available at: https://www.jtcvs.org/article/S2950-6050(25)00026-9/fulltext.
