# Supplementary figures and images for: Distal reentry as a functional primary entry: Insights from blood flow simulation in residual DeBakey IIIb aortic dissection after thoracic endovascular aortic repair
Source: JTCVS Struct Endovasc. 2025 Aug 29;7:100067. doi: 10.1016/j.xjse.2025.100067 (PMC13244783; doi:10.1016/j.xjse.2025.100067)

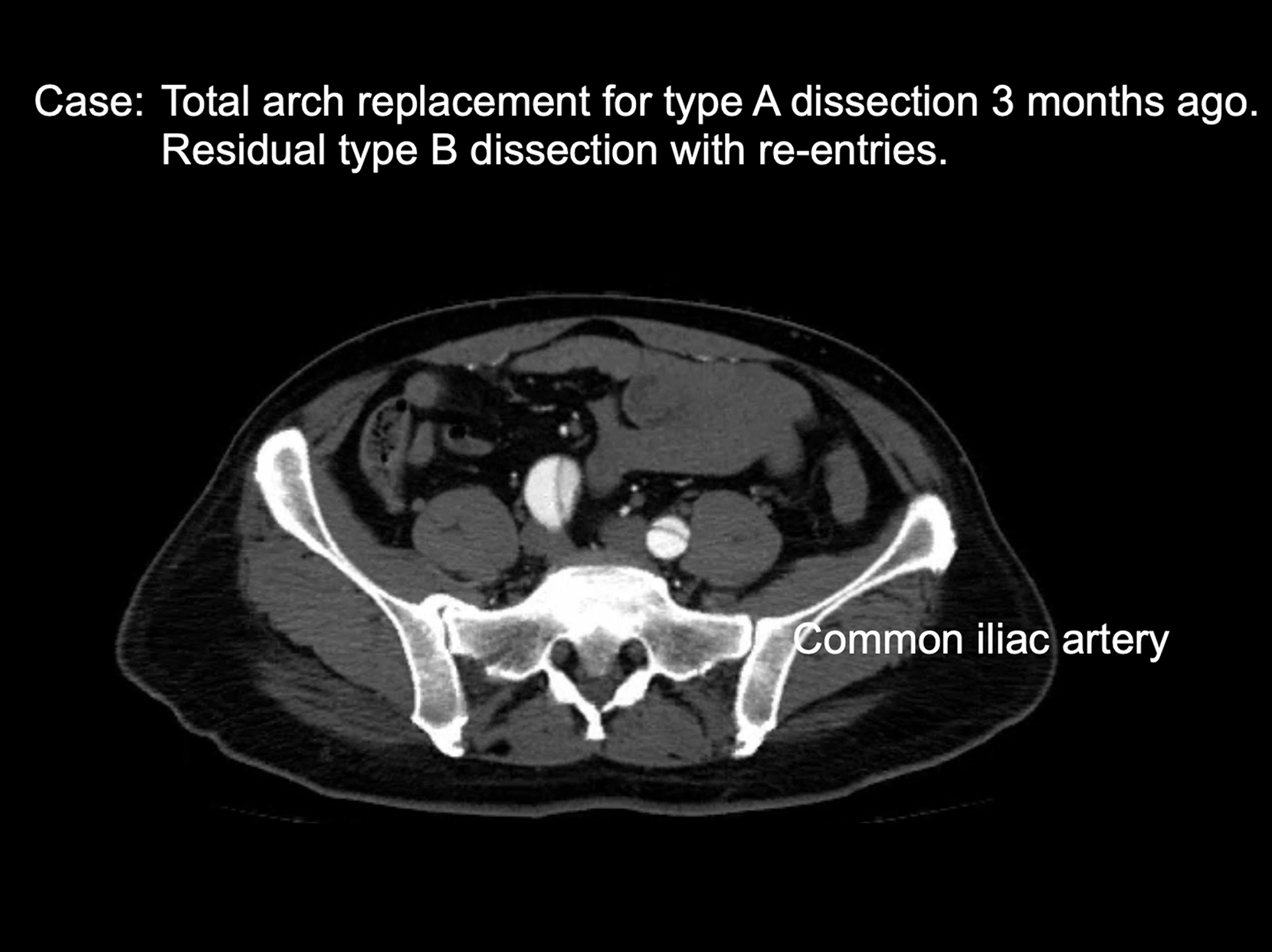

Supplement: Video 1 — Four-dimensional simulation of WSS and blood flow through residual reentries. The video displays the dynamic changes in WSS distribution on the aortic wall throughout a cardiac cycle. It presents 3 consecutive models: (1) after total arch replacement (pre-TEVAR), (2) after the first TEVAR, and (3) after the second TEVAR. The color scale indicates WSS magnitude, with red representing elevated levels. Video available at: https://www.jtcvs.org/article/S2950-6050(25)00026-9/fulltext. [file fx2.jpg]
